# Transient Hypothyroidism: Dual Effect on Adult-Type Leydig Cell and Sertoli Cell Development

**DOI:** 10.3389/fphys.2017.00323

**Published:** 2017-05-23

**Authors:** Eddy Rijntjes, Marcos L. M. Gomes, Nina Zupanič, Hans J. M. Swarts, Jaap Keijer, Katja J. Teerds

**Affiliations:** ^1^Human and Animal Physiology, Department of Animal Sciences, Wageningen UniversityWageningen, Netherlands; ^2^Institut für Experimentelle Endokrinologie, Charité-Universitätsmedizin BerlinBerlin, Germany

**Keywords:** macroorchidism, thyroid hormone, proliferation, testosterone, testis, Leydig cell, Sertoli cell

## Abstract

Transient neonatal 6-propyl-2-thiouracil (PTU) induced hypothyroidism affects Leydig and Sertoli cell numbers in the developing testis, resulting in increased adult testis size. The hypothyroid condition was thought to be responsible, an assumption questioned by studies showing that uninterrupted fetal/postnatal hypothyroidism did not affect adult testis size. Here, we investigated effects of transient hypothyroidism on Leydig and Sertoli cell development, employing a perinatal iodide-deficient diet in combination with sodium perchlorate. This hypothyroidism inducing diet was continued until days 1, 7, 14, or 28 postpartum (pp) respectively, when the rats were switched to a euthyroid diet and followed up to adulthood. Continuous euthyroid and hypothyroid, and neonatal PTU-treated rats switched to the euthyroid diet at 28 days pp, were included for comparison. No effects on formation of the adult-type Leydig cell population or on Sertoli cell proliferation and differentiation were observed when the diet switched at/or before day 14 pp. However, when the diet was discontinued at day 28 pp, Leydig cell development was delayed similarly to what was observed in chronic hypothyroid rats. Surprisingly, Sertoli cell proliferation was 6- to 8-fold increased 2 days after the diet switch and remained elevated the next days. In adulthood, Sertoli cell number per seminiferous tubule cross-section and consequently testis weight was increased in this group. These observations implicate that increased adult testis size in transiently hypothyroid rats is not caused by the hypothyroid condition *per se*, but originates from augmented Sertoli cell proliferation as a consequence of rapid normalization of thyroid hormone concentrations.

## Introduction

It is generally accepted that postnatal development of the testis is highly dependent on the coordinated growth and differentiation of both somatic (Leydig and Sertoli) cells and germ cells (Picut et al., 2015). Beside the gonadotropic hormones luteinizing hormone (LH) and follicle-stimulating hormone (FSH), thyroid hormones (THs) have been implicated to play an important role in testicular, and more particular in Leydig and Sertoli cell development (Gao et al., 2014).

The relation between THs and testis development has mostly been studied by neonatal, transient administration of the TH 3,3′,5-triiodo-L-thyronine (T_3_), inducing hyperthyroidism (van Haaster et al., 1993), or by neonatal, transient administration of the goitrogen 6-propyl-2-thiouracil (PTU), inducing hypothyroidism (Cooke and Meisami, 1991; Cooke et al., 1991; Kirby et al., 1992; van Haaster et al., 1992; Mendis-Handagama et al., 1998; Teerds et al., 1998). Initially it was reported that under PTU-induced hypothyroid conditions, adult-type Leydig cell progenitor formation was arrested in rats up to 21 days after birth (Mendis-Handagama et al., 1998). Following cessation of the hypothyroid condition at the age of 26 days, developing Leydig progenitor cells start to proliferate massively (Hardy et al., 1996), leading to an ~70% increase in the adult-type Leydig cell population in adulthood as compared to the euthyroid controls (Cooke et al., 1991; Hardy et al., 1993). The increased testis weights in adulthood after neonatal PTU treatment are explained by an augmented daily sperm production due to an increased number of Sertoli cells (van Haaster et al., 1992, 1993; Hess et al., 1993; Cooke et al., 1994; Auharek and De Franca, 2010). Sertoli cells are responsible for initiation and progression of spermatogenesis in the testis (Roosen-Runge, 1969; Wagner et al., 2008), and determine testis size as they can only support a certain number of germ cells (Petersen and Soder, 2006). Normally Sertoli cells divide only during a relatively brief phase of testicular development, beginning in fetal life and in rodents lasting until 2–3 weeks postpartum (pp) (Orth et al., 1988; Picut et al., 2015). Hypothyroidism has been reported to prolong the phase of Sertoli cell proliferation, leading to an augmented testicular Sertoli cell number (van Haaster et al., 1992).

Indications that effects of THs on testicular development are at least partially mediated by thyroid hormone receptor α1 (TRA1) come from studies in *Tra1* knockout mice showing a testicular phenotype that has similarities to PTU-treated mice, while the phenotype of *Trb1* knockout mice is not different from wild type mice (Holsberger et al., 2005). Mice with a Sertoli cell specific dominant-negative expression of *Tra1* display enhanced Sertoli cell proliferation and increased testis weight in adulthood (Fumel et al., 2012). The combination of Leydig cell and Sertoli cell dominant-negative *Tra1* expression did not affect steroidogenic activity (Fumel et al., 2015), confirming that Tra1 is the functional TR in Sertoli cells, but not in Leydig cells.

Another method to induce hypothyroidism is by feeding rats a diet low in iodide to deplete endogenous iodine storage alone or in combination with sodium perchlorate to additionally block thyroidal iodide uptake (Crissman et al., 2000; Rijntjes et al., 2009). The advantage of low iodide diets is that the hypothyroid condition can already be induced during fetal life and continued into adulthood, and that perchlorate, unlike PTU, does not interfere directly with the deiodinases, which locally (in-) activate THs (Kuiper et al., 2005). Adult-type Leydig cells in rats on a low iodide diet do differentiate, though this developmental process is delayed and prolonged in comparison to euthyroid control animals. Quite surprisingly, dietary-induced chronic hypothyroidism does not lead to an absolute increase in testis size in adulthood to the same extend, as is the case in the PTU studies (Cooke and Meisami, 1991; Crissman et al., 2000; Rijntjes et al., 2009). The aim of the present study is therefore to elucidate whether these contradicting effects of hypothyroidism are due to the way the hypothyroid conditions are induced (low iodide plus perchlorate vs. PTU) or the length of the intervention (transient vs. chronic). These data will further extend our understanding of the role of TH in testicular development. This is relevant as overt hypothyroidism occurs in 0.3–0.5% of pregnancies (De Groot et al., 2012) and may influence testicular development in boys after birth.

In the underlying investigation, the effects of transient dietary-induced perinatal and PTU-induced neonatal hypothyroidism on gonadal development are studied by restoring the euthyroid status of the rats at several critical time-points in testicular development. A continuously euthyroid and a continuously hypothyroid group are included as reference groups. The development of the testis, with specific emphasis on Leydig and Sertoli cell development, is analyzed, and related to the endocrine condition of the rats (as depicted by the measurement of plasma TSH, T_3_, thyroxine (T_4_), FSH, LH, and testosterone concentrations). We hypothesize that the timing of the switch from a hypothyroid to a euthyroid status is essential for the impact of normalization of TH concentrations on somatic cell development in the testis. Based on previous observations we assume that when this normalization of TH concentrations takes place just prior to puberty, it may induce an instant increase in proliferation of the developing Sertoli cells and immature adult-type Leydig. This will eventually lead to an increase in Leydig cell and Sertoli cell numbers, and consequently increased testicular weight in adulthood.

## Materials and methods

### Chemicals and antibodies

All chemicals were purchased from Sigma (Zwijndrecht, the Netherlands) unless indicated otherwise. The polyclonal antibody against 3β hydroxysteroid dehydrogenase (HSD3B) was a kind gift from the late Dr. Payne (Stanford, CA, USA). Biotinylated horse-anti-mouse antibody, alkaline phosphatase goat-anti-rabbit antibody, and Vectastain ABC-kit Elite were purchased from Vector Laboratories (Burlingame, CA, USA). Mouse-anti-bromodeoxyuridine (BrdU) and sheep-anti-BrdU antibodies were obtained from Beckton and Dickinson (lot nr 10100, Mountain View, CA, USA) and Abcam (ab1893, Cambridge, England), respectively. Polyclonal anti-SOX9 rabbit antibody was purchased from Millipore (lot nr 2065999, Temecula, CA, USA), whereas the secondary fluorescent antibodies anti-mouse Alexa Fluor 488 and anti-rabbit Alexa Fluor 594 were obtained from Abcam; DAPI was obtained from Sigma (D9564, St. Louis, MO, USA). Acetylated BSA (BSA-c) was purchased from Aurion (Wageningen, the Netherlands). The radio-immuno assay (RIA) kits for determination of T_3_, T_4_, and testosterone were obtained from DSL (Webster, TX, USA). SACcel, the secondary donkey-anti-rabbit antibody complex used in the in-house RIA analyses, was obtained from Welcome Reagents (Beckenham, UK.).

### Animals and treatment

The Animal Welfare Committee of Wageningen University approved the animal experiments described in this investigation. Wistar WU (HsdCpbWU) rats were obtained from Harlan (Horst, The Netherlands) at the age of 8 weeks (females) or 10 weeks (males). Female rats were housed individually after arrival. Room temperature (20.5–21.5°C), humidity (55–65%), and light regimen (60–80 lux, light on from 03:00 to 17:00 local daylight saving time) were controlled. Cage enrichment was provided in the form of 10 × 0.4 cm sisal ropes. Two weeks after arrival the female rats of the experimental group were put on an iodide poor diet based on AIN 1993 requirements (Research Diet Services, Wijk bij Duurstede, the Netherlands; Reeves et al., 1993; Schroder-van der Elst et al., 1998), supplemented with 0.75% sodium perchlorate in the drinking water to deplete endogenous iodide stores (Rijntjes et al., 2009). The control group was at the same time put on drinking water without sodium perchlorate and euthyroid control diet, consisting of the iodide poor diet supplemented with 7 μg iodide per 100 g dry weight of diet to fulfill normal iodide requirements of rats. At the age of 12 weeks, the rats were mated. Pups were weaned on postnatal day 28. The offspring was group-housed by gender with their littermates. The thyroid status of the dams was checked at arrival, at the time of mating, at parturition and at weaning (Supplementary Table 1).

**Figure 1:**
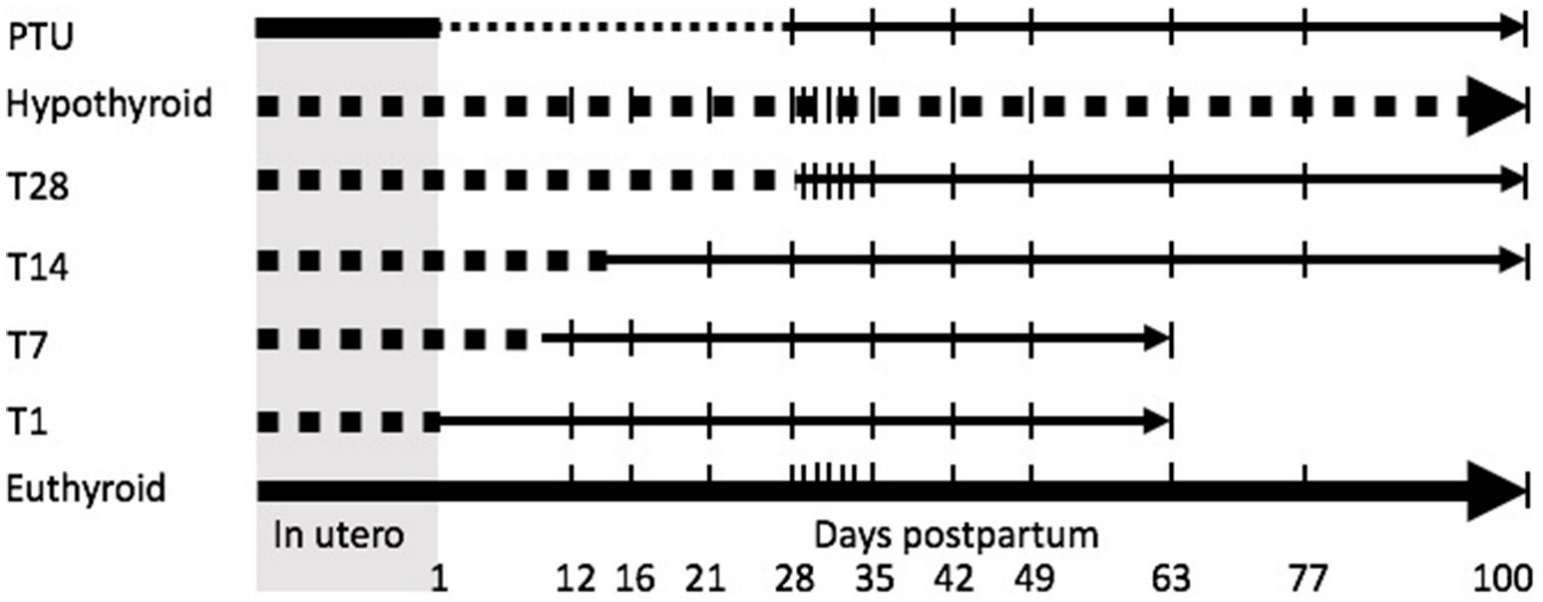
Schematic overview of the experimental design. Dams were treated with an iodide poor diet supplemented with perchlorate for 2 weeks prior to mating to induce hypothyroidism. The diet switched to a control diet (euthyroidism is restored) on the day of birth (T1) or 7 (T7), 14 (T14), or 28 (T28) days postpartum. The offspring was sacrificed with regular intervals. A propylthiouracil (PTU; finely dotted line) treated (treatment from day of birth to day 28 postpartum), continuously hypothyroid (iodide poor diet and perchlorate induced; tickly dotted line) and euthyroid group served as controls. | represents a day of sacrifice.

The experimental hypothyroid diet was continued until day 1 pp (T1), 7 (T7), 14 (T14), or 28 (T28) days pp, when the rats were switched to the euthyroid diet (Figure 1). The rats of the T1 and T7 groups were sacrificed at the age of 12, 16, 21, 28, 35, 42, 49, and 63 days pp (*n* = 5–12 per group). The animals of the T14 and T28 groups were sacrificed at the age of 21, 28, 35, 42, 49, 63, 77, and 100 days pp (*n* = 5–12 per group). These different time points were chosen as they represent important stages in Leydig cell and Sertoli cell development under euthyroid conditions, e.g., day 12 pp is the age at which the formation of the adult-type Leydig cell population is started, between days 16 and 21 pp Sertoli cells cease to proliferate, between days 21 and 28 pp adult-type Leydig cell progenitors under euthyroid conditions undergo a wave of proliferation, around day 35 pp immature Leydig cells develop (Hardy et al., 1989). Furthermore, from our previous study we know that adult-type Leydig cell development is delayed under chronic fetal/neonatal hypothyroid conditions, therefore the ages 42, 49, 63, 77, and 100 days pp were included as well (Rijntjes et al., 2009).

To compare our current study to earlier studies using the goitrogen PTU, we included a group of dams on the control diet that received 0.1% PTU in their drinking water from the day the pups were born onwards (group: PTU). PTU treatment was continued up to day 28 pp when the animals were weaned. These animals (*n* = 5–6 per group) were sacrificed at the same ages as the T14 and T28 groups.

In a separate experiment, chronically hypothyroid pups were weaned at day 28 pp, switched to the euthyroid diet and killed on day 29, 30, 31, 32, or 33 pp, respectively (*n* = 5 per group). Age-matched rats that after weaning continued to be fed the iodide poor diet supplemented with perchlorate served as control (*n* = 5–6 per group). These different time points of sacrifice were chosen based on the study by van Haaster et al. (1992) who showed that Sertoli cells under PTU induced hypothyroidism cease to proliferate around the age of 30 days pp, to obtain novel information on the pattern of Sertoli cell proliferation and its consequences for testis size in adulthood.

All rats received a subcutaneous injection with bromodeoxyuridine (BrdU) (150 mg/kg BW) in saline 2 h before sacrifice. BrdU is a compound that is specifically incorporated in the DNA of cells in the S-phase of the cell cycle and gives an indication of the proliferative activity of a tissue.

Rats were anesthetized using carbon dioxide and oxygen (flow: 1:2). Blood was collected by heart puncture (5 IU heparin per ml blood). Rats were killed by decapitation and organs were collected. Plasma was stored at −20°C until further analysis.

### Histology and immunohistochemistry

The testes were fixed in 4% buffered formalin for 24–48 h and stored in PBS at 4°C until further processing. Tissues were dehydrated and embedded in paraffin. The testes were cut in 5 μm sections using a microtome (Reichert-Jung 2030, Heidelberg, Germany). The sections were mounted either on Superfrost or Superfrost Plus slides (Menzel-Gläser, Braunschweig, Germany) and stored overnight at 37°C before PAS (Periodic Acid Schiff)/haematoxylin staining or immunohistochemical staining. All incubations were performed at room temperature, unless stated otherwise.

In order to determine the proliferation of the developing adult-type Leydig cell population, testis sections were double-stained for BrdU and HSD3B and counterstained with Mayer's haematoxylin as described previously (Rijntjes et al., 2008). As a control the primary antibodies were replaced by buffer or by isotype IgG (Vector) at dilutions as indicated by the manufacturer. Progenitor, immature, and mature adult-type Leydig cells were identified by the presence of HSD3B immunoreactivity (blue-stained cytoplasm) and counted in randomly chosen areas in a minimum of three different sections per testis that were at least 100 μm apart. Only those HSD3B positive cells were counted in which the nucleus was present. Proliferating Leydig cells were identified by the presence of BrdU (brown) staining of the nucleus. Per testis, 1,500 Leydig cells (HSD3B labeled and HSD3B plus BrdU labeled) were counted and the percentage of Leydig cells in S-phase was calculated. In case of young animals when less than 1,500 HSD3B positive cells were present, all Leydig cells in at least four testicular cross-sections were counted.

In order to determine the proliferative capacity of Sertoli cells in (transient) hypothyroid rats after weaning, Sertoli cells were identified by presence of SOX9 immunoreactivity. SOX9 belongs to the family of SOX proteins (Sry-related HMG box proteins) that are transcription factors (Fröjdman et al., 2000). It can be reliably used as a Sertoli cell nuclear marker in the pubertal rat testis to discriminate between Sertoli cells and spermatogonia. Therefore, combination of Sertoli cell marker SOX9 with the proliferation marker BrdU, allowed us to identify proliferating Sertoli cells. In short, histological sections were routinely deparaffinized and rinsed in PBS (3 × 5 min; 0.01 M, pH 7.4). Antigen retrieval was performed by microwaving the slides in 0.01 M sodium-citrate buffer (pH 6.0) (3 × 5 min) followed by a cooling down period (30 min). The slides were washed in PBS + 0.5% Triton X-100 (3 × 5 min) and rinsed in PBS (2 × 5 min). Non-specific binding was blocked by incubating sections with 5% normal goat serum (30 min). Sections were incubated with the primary monoclonal anti-BrdU mouse antibody or primary polyclonal sheep-anti-BrdU antibody [both diluted 1:100 in PBS/acytelated BSA (BSAc)] (60 min). After rinsing in PBS (4 × 5 min) sections were incubated with a secondary fluorescent goat anti-mouse antibody (Alexa Fluor 488, diluted 1:200 in PBS/BSAc) (60 min). Next, slides were rinsed in PBS (4 × 5 min) and incubated overnight at 4°C with the primary polyclonal anti-SOX9 rabbit antibody (diluted 1:500 in PBS/BSAc). The following day, sections were washed in PBS (4 × 5 min) and incubated with the secondary fluorescent goat anti-rabbit antibody (Alexa Fluor 546, diluted 1:200 in PBS/BSAc) (60 min). Sections were again rinsed in PBS (4 × 5 min) followed by DAPI staining (1 μg/ml, 5 min). After the final wash in PBS (4 × 5 min) sections were mounted with Vectashield, covered by a coverslip and stored in the dark at 4°C. All antibody incubations were performed in a humid chamber. For neonate animals (12–28 and 29–33 days pp), digital images of 20 round seminiferous tubules per animal with positive BrdU staining were randomly taken; the same tubules were used to detect SOX9-positive cells (200x magnification). The secondary antibody for BrdU is green fluorescent and the secondary antibody for SOX9 is red fluorescent. Overlay of green and red labels resulted in a yellow pattern, indicating proliferating Sertoli cells. Sections were examined with an Axioskop 2 microscope equipped with Colibri and Axiocam MRc 5 camera (Carl Zeiss, Göttingen, Germany).

Periodic acid Schiff (PAS)/haematoxylin stained slides were used to measure the diameter of seminiferous tubules, as well as seminiferous epithelium height in 100-day-old animals, using 20 random tubule's cross sections (stage VIII) per animal. The same seminiferous tubules were used to determine the number of Sertoli cells per tubular cross section. Only Sertoli cells with clear nucleus and prominent nucleoli were considered. All image analyses were made using Axiovision 40 v 4.8.2.0 software (Carl Zeiss).

### Radio immunoassays

RIAs for total T_4_ (DSL-3200), total T_3_ (DSL-3100), 3α-androstanediol-glucuronide (DSL-6000), and testosterone (DSL-4100) were assayed according to the manufactures protocol. LH, FSH, and TSH concentrations were determined by validated in-house double-antibody RIAs for rat serum analysis using materials supplied by the National Institute of Diabetes, Digestive and Kidney Diseases (NIDDK; Bethesda, MD, USA) (Mattheij et al., 1995; Palm et al., 2001a,b). For all in-house RIAs SACcel (donkey anti-rabbit) was used as the secondary antibody. The concentrations of hormones were expressed in terms of NIDDK standards. The detection limits of the assays were: 5 ng/ml for total T_4_, 0.25 ng/ml for total T_3_, 0.5 ng/ml for 3α-androstanediol glucuronide, 0.03 ng/ml for LH, 0.1 ng/ml for testosterone and TSH and 0.4 ng/ml for FSH. The intra- and interassay variation was determined using several pools of rat serum and were less than 11% for all purchased RIAs and less than 9.5% for all in-house RIAs.

### Statistical analysis

Data are expressed as mean ± standard error of the mean (SEM). Previous experiments suggested that testicular development was dependent on the founders; hence per age group not more than one pup per dam was used. Statistical analysis was carried out using SPSS 20.0 for Windows. Data were tested for normality using the Shapiro-Wilk test. If normality could not be assumed all groups were log10 transformed. Means were compared using ANOVA with a Bonferonni or Dunnett T3 as *post-hoc* test. Values of *p* < 0.05 were considered to be significantly different.

## Results

The hypothyroid condition of the dams did not affect litter size and gender ratio of the offspring (data not shown). Body weight, testis weight, plasma TSH, total T_4_, total T_3_, testosterone, LH, and FSH concentrations from transiently (T1, T7, and T14) hypothyroid rats did not differ from euthyroid controls. Detailed data on these variables from day 12 to 21 pp is provided in Supplementary Table 2.

**Figure 2:**
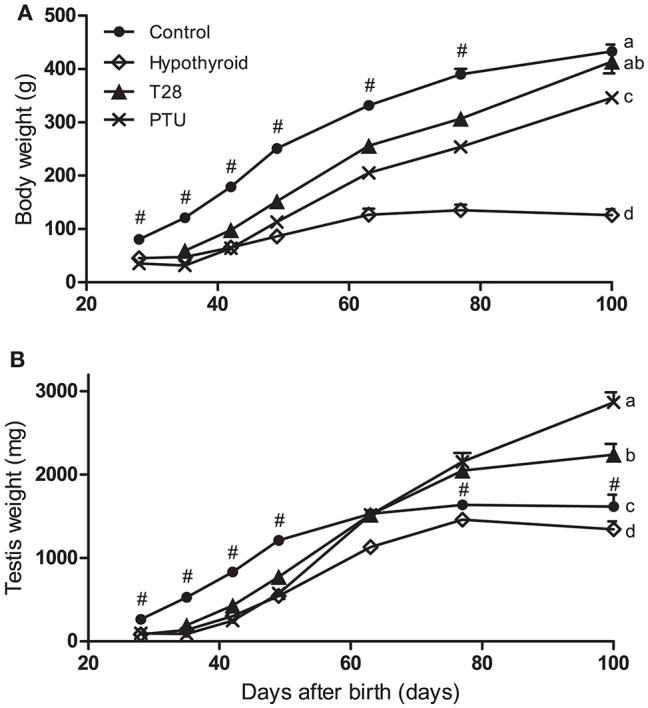
Body **(A)** and testis **(B)** weights of euthyroid control, continuously hypothyroid and transiently (T28/PTU-treated) hypothyroid rats. The body weights of the euthyroid control rats are significantly higher for the entire experimental period (*p* < 0.05). Testis weights of T28 and PTU rats were significantly lower compared to the euthyroid controls up to day 49 pp, caught up by day 63 pp, to become significantly higher 2 weeks later (*p* < 0.05). Testis weights of the continuously hypothyroid animals are identical to the euthyroid controls at day 100 pp. Values represent means + SEM. # indicates a significant different from the euthyroid control group to all other groups (*p* < 0.05). Groups that do not share the same letter differ significantly on day 100 pp, *n* = 5–12.

Except for the T28 rats on day 100 pp, when there was no difference, the body weights of the hypothyroid, T28 and PTU pups were significantly lower at all ages when compared to the respective age-matched euthyroid controls. The body weights of the T28 and PTU groups were significantly higher than those of the continuously hypothyroid animals from day 49 and 63, respectively, onwards (Figure 2A).

### Testis weight

Chronic hypothyroidism resulted in lower testis weights in adulthood. The T28 and PTU rats showed a marked increase in testis weight from day 42 pp onwards. By the age of 63 days all transiently hypothyroid groups had testis weights comparable to the euthyroid control group whereas they concurrently differed from continuously hypothyroid rats. The testis weight from the PTU and T28 groups was significantly higher from day 77 onwards compared to euthyroid control rats (Figure 2B).

### Thyroid hormone status

**Figure 3:**
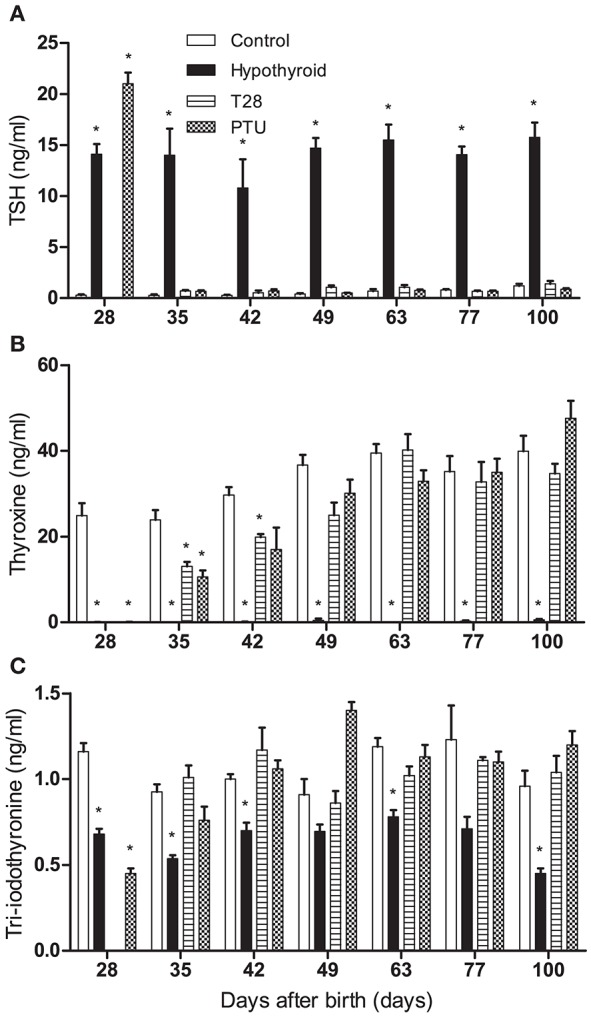
Plasma thyroid stimulating hormone (TSH) **(A)**, total thyroxine (T_4_) **(B)**, and total tri-iodothyronine (T_3_) **(C)** concentrations of euthyroid control, continuously hypothyroid and transiently (T28/PTU-treated) hypothyroid rats. T_3_ and T_4_ levels are decreased in hypothyroid animals. After the switch to a euthyroid control diet, TSH values recover rapidly, whereas T_3_ and T_4_ levels follow soon after. Bars represent means + SEM. ^*^Indicates a significant difference from the control group (*p* < 0.05), *n* = 5–8.

Plasma TSH, T_4_, and T_3_ concentrations were determined to assess the thyroid status of the rats (Figures 3A–C). At the time of pregnancy onset and parturition the hypothyroid dams had significantly increased TSH concentrations and decreased T_4_ concentrations (see Supplementary Table 1), implicating that the developing fetuses were exposed to a hypothyroid environment. By day 21 pp, the thyroid status of the T1, T7, and T14 dams were undistinguishable from euthyroid controls.

Plasma TSH concentrations in the hypothyroid offspring were significantly increased compared to age-matched euthyroid controls at all ages. TSH concentrations of rats on PTU on day 28 pp were even higher than those on an iodide poor diet combined with perchlorate. All TSH concentrations were restored to control levels within 1 week after cessation of the hypothyroid diet or PTU treatment (Figure 3A). In contrast, plasma T_4_ concentrations continued to be decreased 1 week after the switch to the euthyroid diet on day 28, although the concentrations were significantly higher than those of the continuously hypothyroid rats (Figure 3B). A detailed analysis of plasma from the T28 rats sacrificed with daily intervals from day 29 to 32 revealed that the T_4_ concentrations came up to euthyroid control concentrations (27–32 ng/ml) on day 29 (46 ± 27 ng/ml) and day 30 (22 ± 6 ng/ml)pp, but then significantly fell back below euthyroid controls on day 31 (11 ± 2 ng/ml) and day 32 (6 ± 2 ng/ml) pp. Similar to TSH concentrations, plasma T_3_ concentrations were within the euthyroid control range within 1 week after both goitrogen treatments was discontinued (Figure 3C). Detailed analysis of the T_3_ concentration of plasma from T28 rats sacrificed from day 29 to 32 pp revealed no significant differences from the euthyroid controls, indicative of a tight regulation of the plasma T_3_ concentrations.

### Testis development

**Figure 4:**
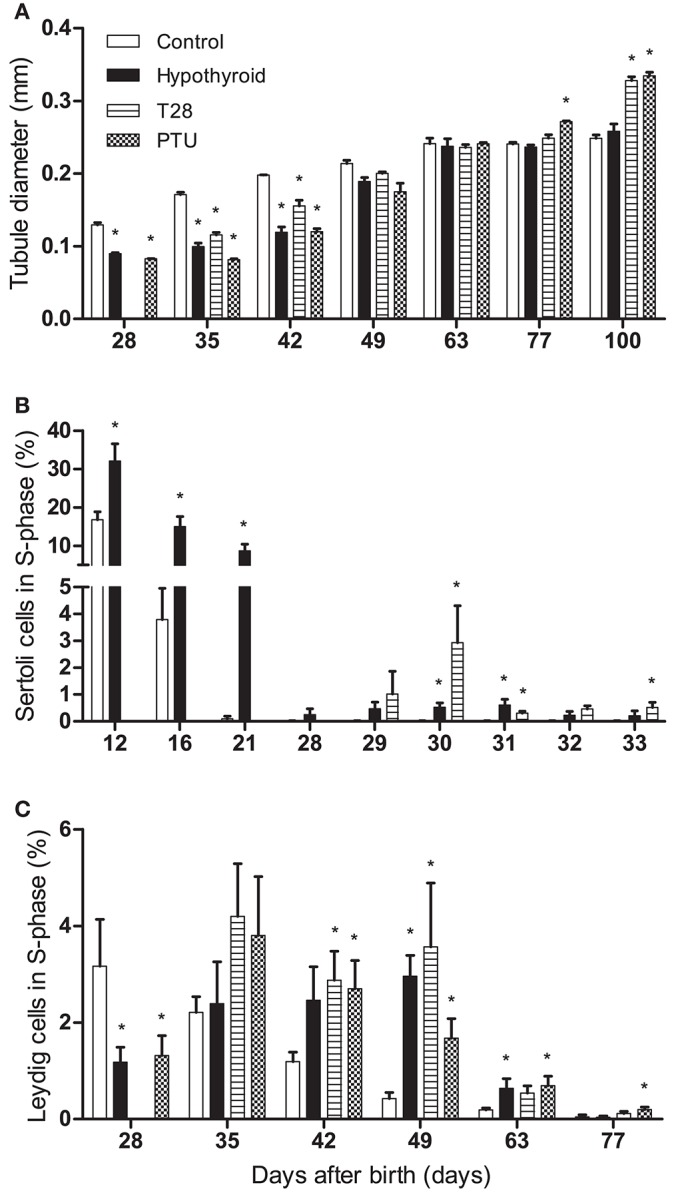
Seminiferous tubule diameter (in mm) **(A)**, percentage of BrdU labeled Sertoli cells **(B)**, and percentage of BrdU labeled Leydig cells **(C)** in euthyroid control, continuously hypothyroid and transiently (T28/PTU-treated) hypothyroid rat testes. Testes were double stained using BrdU and SOX9 (Sertoli cells) or HSD3B (Leydig cells) as markers. The Sertoli cells in the euthyroid control group already stopped proliferation well-before day 29 pp. Bars represent means + SEM. ^*^Indicates a significant difference from the control group (*p* < 0.05), *n* = 5–8.

To evaluate Sertoli cell development under transient hypothyroid conditions, seminiferous tubule lumen formation, and tubule diameter were evaluated. In the rat, initiation of tubule lumen formation, a marker for Sertoli cell differentiation (van Haaster et al., 1993), typically starts between day 15 and 17 pp, to be completed by day 28 pp. Continuous hypothyroidism led to a delay in lumen formation (below 9% of the tubules have a lumen by day 28 pp, whereas 83% of the seminiferous tubules showed opening in the control group by day 21 pp), but was completed by day 42 pp. The T28 rats completed lumen formation by day 35 pp, the PTU treated animals however, completed lumen formation at day 42 pp, showing an even further delay compared to the T28 rats. The diameter of the seminiferous tubules, which gives information about the progression of spermatogenesis, was significantly smaller in all groups compared to the euthyroid control up to day 42 pp (Figure 4A). From day 77 pp onwards, the seminiferous tubule diameter was larger in PTU rats, matching the findings of larger testicular weights. By the age of 100 days the seminiferous tubule diameter was also significantly larger in the T28 rats, coinciding with augmented Sertoli cell numbers per tubule cross section and larger tubule epithelial height (Table 1, Figure 4A).

**Table 1:** Epithelial height and Sertoli cell numbers in cross sections of seminiferous tubules at the age of 100 days pp of euthyroid control, continuously hypothyroid, and transiently hypothyroid (T28/PTU-treated) rats.

**Age: 100 days pp**	**Mean Sertoli cell number**	**Mean epithelial height in μm**
Control	18 ± 1	67.6 ± 2.4
Hypothyroid	23 ± 1	68.7 ± 2.6
T28	29^*^±2	79.6^*^±2.1
PTU	29^*^±1	85.0^*^±2.0

*Seventy-two days after discontinuation of the hypothyroid condition, the Sertoli cells numbers per tubule cross section and seminiferous epithelial height are increased, compared to the euthyroid controls and chronic hypothyroid rats. Values represent means ± SEM*.

* *Indicates a significant difference from the control group (p < 0.05), n = 5*.

**Figure 5:**
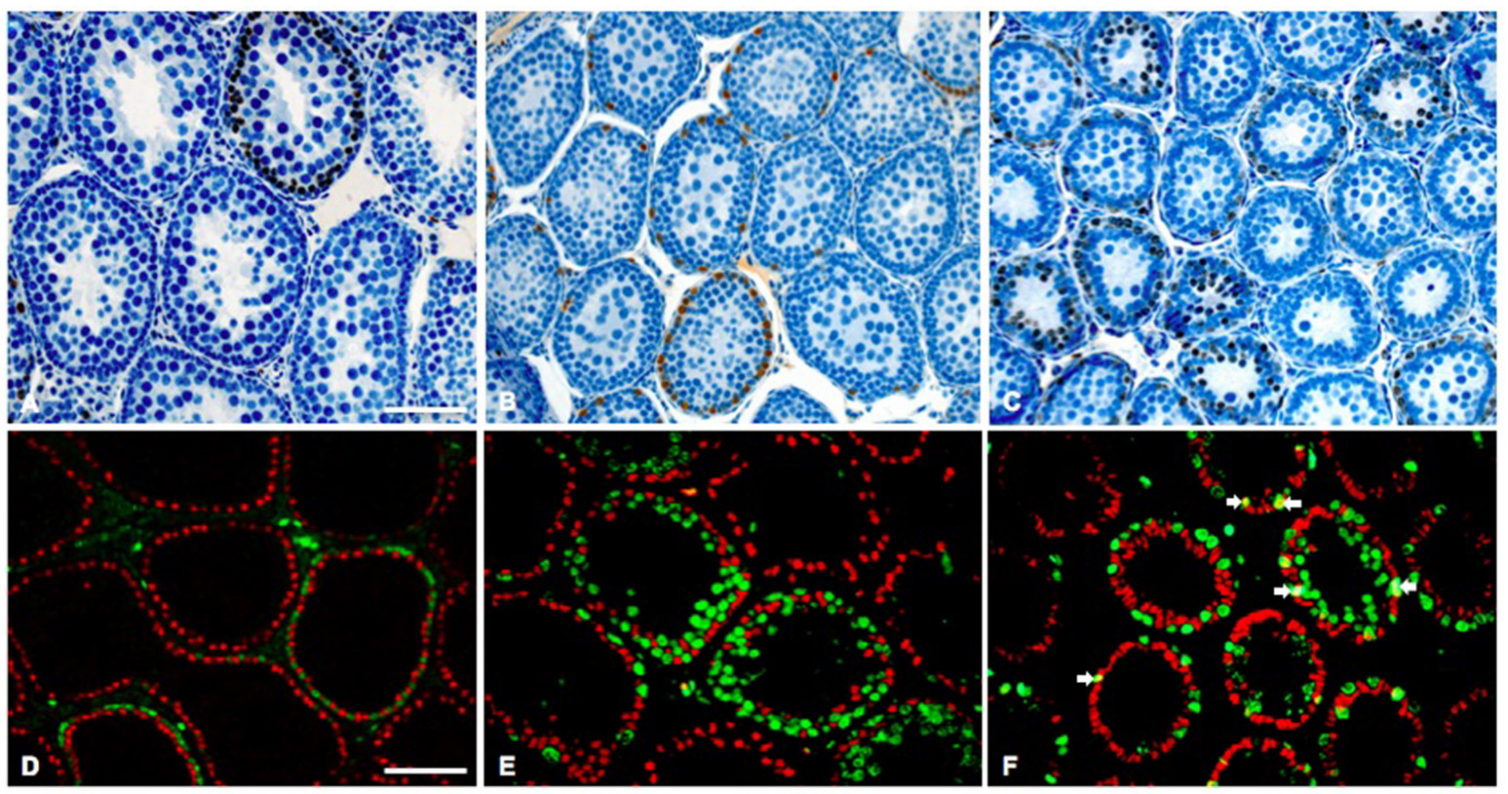
Representative histological testis sections of 30-day-old rat testes. **(A–C)** BrdU (brown nuclei)/hematoxylin staining of a euthyroid control **(A)**, chronic hypothyroid **(B)**, and transient hypothyroid (T28) rat testis. **(D–E)** Fluorescent staining for SOX9 (red nuclei) and BrdU (green nuclei) of a euthyroid control **(D)**, chronic hypothyroid **(E)**, and transient hypothyroid (T28) rat testis **(F)**. In the transient (T28) testis several SOX9/BrdU positive Sertoli cells were observed (arrows). Bar **(A–C)** represents 30 μm, bar **(D–E)** represents 40 μm.

To obtain an impression of the proliferation profile of Sertoli cells under hypothyroid conditions and after the alleviation of the hypothyroid status at day 28 pp (T28 group), BrdU incorporation in SOX9 positive Sertoli cells was used to determine their proliferative capacity between days 12 and 33 pp (Figures 4B, 5). Sertoli cells had ceased to proliferate in euthyroid control rats by the age of 28 days pp. After discontinuation of the dietary intervention in the T28 group Sertoli cell proliferation increased, reaching peak levels within the timeframe studied by day 30 pp, 2 days after the switch to the euthyroid diet (Figure 4B).

BrdU incorporation in HSD3B positive Leydig cells was used to evaluate Leydig cell proliferation (Figure 4C). With the differentiation of the progenitor Leydig cells into immature-type Leydig cells by the age of 28–35 days pp, Leydig cell proliferation goes down in euthyroid control rats. Leydig cell proliferation in T28 and PTU rats showed a pattern comparable to the continuously hypothyroid rats, although at a slightly higher level. Leydig cell proliferation for these groups only came down from their peak levels after day 49 pp.

### Reproductive hormone concentrations

**Figure 6:**
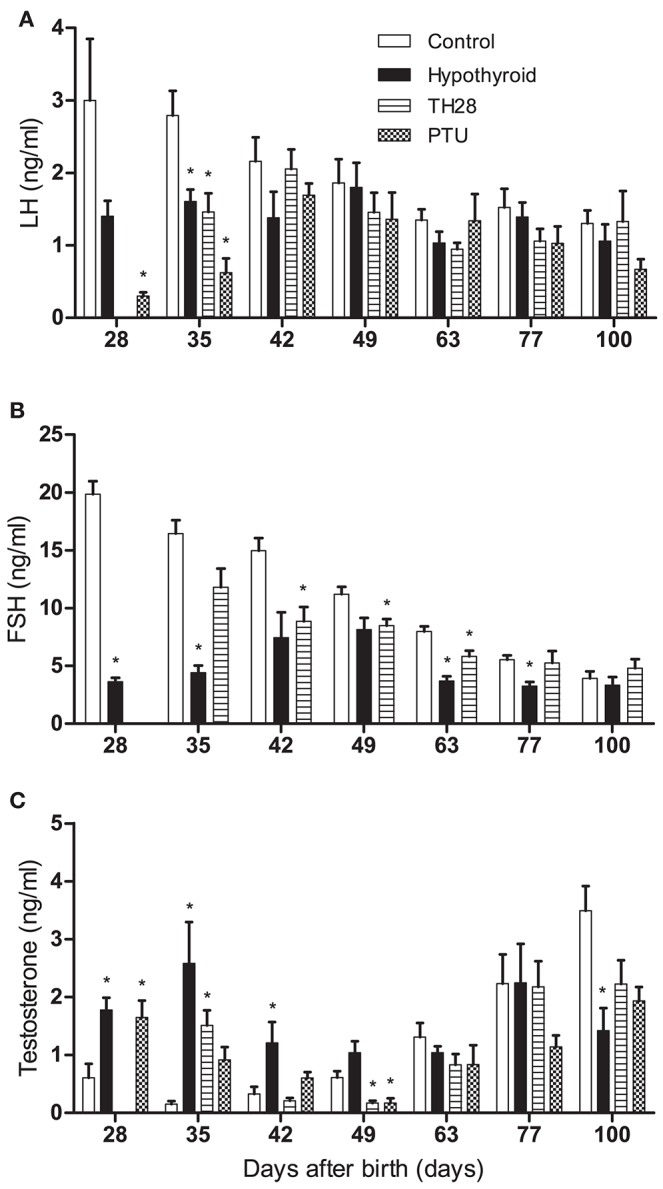
Plasma luteinizing hormone (LH) **(A)**, follicle stimulating hormone (FSH) **(B)**, and testosterone **(C)** concentrations in euthyroid control, continuously hypothyroid and transiently (T28/PTU-treated) hypothyroid rats. FSH concentrations have not been determined in PTU treated rats. Bars represent means + SEM. ^*^Indicates a significant difference from the control group (*p* < 0.05), *n* = 5–8.

Testicular development is under the influence of the gonadotropins LH and FSH. In parallel to continuously hypothyroid rats, plasma LH concentrations were lower in T28 and PTU animals on day 28–35 pp, as compared to the euthyroid controls (Figure 6A). Concomitantly FSH concentrations of the T28 group were also lower up to day 63 pp (Figure 6B). Concurrent with reduced plasma LH concentrations in the continuously hypothyroid and T28 groups, plasma testosterone concentrations were significantly higher at day 28 and 35 pp as compared to the age-matched euthyroid controls. Where the testosterone concentrations were initially higher in the T28 and continuously hypothyroid rats, at day 49 pp testosterone concentrations were higher in the euthyroid control rats compared to the T28 and PTU treatment groups. By day 100 pp there was no longer a difference in testosterone concentrations between the euthyroid controls and the T28 and PTU groups (Figure 6C). For the developing immature-type Leydig cell, 3α-androstanediol is the main steroid produced in the testis (Tapanainen et al., 1984). Its glucuronide was determined in the control and hypothyroid rats from day 16 onwards. 3α-androstanediol-glucuronide was decreased on day 16 pp in the hypothyroid rats compared to the controls (3.2 ± 0.2 vs. 4.5 ± 0.4 ng/ml, *p* = 0.007), and showed no difference in the days thereafter.

## Discussion

In contrast to transient PTU induced neonatal hypothyroidism (van Haaster et al., 1992; Hess et al., 1993; Joyce et al., 1993; Teerds et al., 1998), dietary induced chronic fetal-postnatal hypothyroidism nor chronic PTU induced neonatal hypothyroidism lead to a higher absolute testis weight in adulthood (Meisami et al., 1994; Rijntjes et al., 2009). In the present study, we investigated the possible cause of these apparently contradicting observations by restoring the euthyroid status of prepubertal rats at several critical time-points during Sertoli cell and Leydig cell development. If the hypothyroid condition is discontinued at 28 days pp, some striking changes are observed; the plasma concentration of T_3_ is back at the euthyroid control concentrations within a day while T_4_, after a short peak just after discontinuation of the low iodide/perchlorate treatment, takes a few weeks to fully return to euthyroid control levels. The testis weights of the T28, but not T1, T7, or T14 rats, follow a similar pattern to that of transiently PTU treated (1–24 days pp) rats (Kirby et al., 1992). Surprisingly, within 2 days after cessation of our dietary intervention in the T28 group, Sertoli cell proliferation shows a sharp, significant increase and remains elevated up to day 33 pp. Although in continuously hypothyroid rats Sertoli cells are also still actively proliferating, the percentage of BrdU positive cells is significantly lower compared to the T28 animals, offering at least a partial explanation for the absence of an augmented testis weight in the chronic hypothyroid group in adulthood.

Both thyroid hormones, T_3_ and T_4_, show an immediate bounce to euthyroid control concentrations after the discontinuation of administration of the goitrogen to a low iodide diet. Similar findings have been made in an earlier study, in which 2 days after interruption of PTU treatment, serum T_3_ concentrations in outbred male CD rats had normalized, with a peak in T_3_ levels about 50% above controls on day 3 and T_4_ following shortly after (Cooper et al., 1983). The initial systematic clearance of perchlorate and PTU is rapid with a half-life in sera below 8 h (Cooper et al., 1983; Yu et al., 2002). The iodide entering the thyroid is now quickly incorporated in the precursors of TH, mono- and diiodothyrosine. In case of discontinuation of perchlorate treatment the iodide can be found in especially T_3_ and to a lesser extend in T_4_ within a day (Studer and Greer, 1967). As the iodide availability and thyroid function are restored, the thyrocyte will shift its production from T_3_ to T_4_, the latter which did not normalize after an initial bounce within 7 days.

In euthyroid rats Sertoli cells stop to proliferate between days 16 and 21 pp, when differentiation is initiated (Orth et al., 1988; Picut et al., 2015), a process that is advanced under hyperthyroid conditions (van Haaster et al., 1993). Under PTU induced neonatal hypothyroid conditions the timeline of Sertoli cell proliferation and differentiation seems to be disturbed (van Haaster et al., 1992, 1993; Hess et al., 1993; Cooke et al., 1994; Auharek and De Franca, 2010). The phase of Sertoli cell proliferation is prolonged and consequently the initiation of Sertoli cell differentiation is delayed; Sertoli cells cease to proliferate between days 30 and 36 pp, 4–10 days after PTU treatment is discontinued. As the final size of the testis is determined by the number of Sertoli cells present (Berndtson and Thompson, 1990) and Sertoli cells can only nurse a fixed number of germ cells (Orth et al., 1988), it is not surprising that under PTU induced transient hypothyroid conditions, Sertoli cell number and testis size in adulthood is increased compared to the untreated euthyroid animals (Cooke and Meisami, 1991; Cooke et al., 1991; Hess et al., 1993; Simorangkir et al., 1995). The present study in which hypothyroidism is induced perinatally by dietary intervention and continued until sacrifice confirms that hypothyroidism leads to a prolonged period of Sertoli cell proliferation and a delay in Sertoli cell differentiation as depicted by a delay in tubule lumen formation, a marker for Sertoli cell differentiation (van Haaster et al., 1992, 1993; Hess et al., 1993; Cooke et al., 1994; Auharek and De Franca, 2010). It was expected that this elevated Sertoli cell proliferation between days 12 and 28 pp in the chronic hypothyroid group would, like in case of PTU-induced hypothyroidism (van Haaster et al., 1992), lead to an increase in Sertoli cell number per tubule cross section in adulthood as well as a significant increase in testis weight. This however is not the case; seminiferous tubule diameter, Sertoli cell number per tubule cross section as well as the testis weight of the chronic hypothothyroid animals is not different from the euthyroid control group. One can argue that the absence of an increased testis weight in may be due to the fact that part of the Sertoli cells have undergone degeneration between days 28 and 100 postpartum as a consequence of the continuous exposure to reduced thyroid hormone level. Although this may offer and explain for the observation why Sertoli cell numbers per seminiferous tubule cross section and testis weight in the chronic hypothyroid and euthyroid control animals are comparable, we do not think the case. We have examined testicular development in the chronic hypothyroid animals carefully on a weekly basis. If indeed in the chronic hypothyroid testes Sertoli cell degeneration had occurred, this should have led to a disturbance in spermatogenesis, as spermatocytes and spermatids are highly dependent on Sertoli cells for their development and survival. Except for a delay in the completion of the first cycle of spermatogenesis, we have not observed any other abnormalities in the process of spermatogenesis in the chronic hypothyroid animals.

A more detailed analysis of Sertoli cell proliferation in the days following discontinuation of the hypothyroid condition at day 28 pp as performed in the present study, shows that within 2 days after the dietary intervention is stopped, Sertoli cell proliferation is increased 6- to 8-fold compared to the continuously hypothyroid rats. Although we nor any other investigators have analyzed Sertoli cell proliferation in the PTU-treated animals group in the first days after discontinuation of PTU treatment, we assume based on the study by van Haaster et al. (1992) that a comparable wave of Sertoli cell proliferation occurs. This assumption is based on the fact that the thyroid status of the animals in the PTU group and the T28 group is comparable as well as the percentages of BrdU labeled Sertoli cells 4 days after discontinuation of PTU treatment (van Haaster et al., 1992) and on day 32 pp in the T28 group. Based on these results we therefore hypothesize that it is not the hypothyroid condition itself but the switch from a hypothyroid to an euthyroid diet that is responsible for an additional wave in Sertoli cell proliferation leading to an increased number of Sertoli cells per seminiferous tubule cross section in adulthood and thus in an increased testis weight. This assumption is further supported by the significant increase in Sertoli cell numbers per tubule cross section in the T28 group that already becomes apparent at day 31 pp. The mechanism behind the enhanced Sertoli cell proliferation following cessation of the hypothyroid condition is not clear. One of the candidate factors involved may be testosterone. At day 28 pp at the time of discontinuation of the hypothyroid condition, the expression of the AR in Sertoli cells is negligible. However, the switch to a euthyroid diet leads to a rapid induction of AR expression in Sertoli cells of the T28 group, implicating that testosterone may play a role (Supplementary Figure 1, Rijntjes et al, unpublished observations).

We are aware that Sertoli cell proliferation in the T28 group between days 29 and 31 pp is only 6- to 8-fold higher compared to the chronic hypothyroid rats and thus one can argue that this increase is not sufficient to account for the increased Sertoli cell numbers in adulthood. However, one must keep in mind that we provided data with 24 h intervals for the T28 and chronic hypothyroid groups. Along these intervals animals were exposed to the cell proliferation marker BrdU for only 2 h. Sertoli cell proliferation is not considered to be a synchronized process, implicating that before and after the period of BrdU administration other Sertoli cells will be entering and leaving the S-phase of the cell cycle. Consequently, Sertoli cells that are in the later stage of the G1 phase of the cell cycle, or have already passed the S-phase and are in the G2 phase will not be labeled by BrdU. We therefore cannot exclude that a potentially higher peak in proliferation might have been missed between days 29 and 30 or 30 and 31 pp (at the time thyroid hormone concentrations are normal). These cells will contribute to the proliferative capacity of the Sertoli cell population as well and thus to the observed increase in Sertoli cell number per seminiferous tubule cross section and testis weight in the T28 group at day 100 postpartum.

In contrast to the above observations, if the hypothyroid condition is discontinued at day 7 or 14 pp, TH concentrations return to the euthyroid control levels within 7 days and no effect on testis weight is observed in adulthood at the age of 63 days. Apparently the increased Sertoli cell proliferation observed in the hypothyroid rats on day 12 pp does not seem to contribute significantly to the final size of the Sertoli cell population in adulthood. This observation is in contrast to the study by Cooke et al. (1992) who induced hypothyroidism in newborn male rats by PTU treatment and showed that if the hypothyroid condition was discontinued at day 8 or 16 pp, testis weight was significantly increased by the age of 90 days. At present, we do not have an explanation for this discrepancy, except for the different ways hypothyroidism was induced (prenatal vs. postnatal; perchlorate in combination with a low iodide diet vs. PTU).

There are several lines of evidence that implicate that THs are essential for the development of a normal adult Leydig cell population. For example, Leydig cell progenitor development is delayed in hypothyroid neonatal rats (Teerds et al., 1998; Rijntjes et al., 2009). The first progenitor cells are identified at day 16 pp, 4 days later than in the euthyroid controls, which suggests that the reduced THconcentrations are still sufficient to make stem Leydig cell differentiation possible (Rijntjes et al., 2009). Only on day 16 pp, the 3α-androstanediol-glucuronide concentration in the hypothyroid rat is decreased; as also in the euthyroid control rats the levels go down with age. In a study using methimazole to induce hypothyroidism, an even more substantial decrease was noted, but then on day 21 pp (Cristovao et al., 2002). As 3α-androstanediol is the main steroid produced by the newly developing immature adult-type Leydig cell (Tapanainen et al., 1984), these reduced levels suggest a delay in the differentiation of the Leydig cells at this period of development. In contrast, in the presence of elevated neonatal T_3_ concentrations the formation of Leydig cell progenitors is advanced (Teerds et al., 1998; Ariyaratne et al., 2000; Mendis-Handagama et al., 2007). In line with these observations, an *in vitro* study has shown that undifferentiated stem Leydig cells are unable to differentiate into HSD3B positive androgen producing Leydig cell progenitors in the absence of T_3_ (Ge et al., 2006). The present study provides further proof to these observations, because when the hypothyroid diet is discontinued before or at day 14 pp, Leydig cell progenitor proliferation follows the same profile as the euthyroid control rats, suggesting a rapid catch-up after return to the euthyroid condition. When the hypothyroid condition is discontinued at day 28 pp, the percentage of Leydig cells in S-phase of the cell cycle in the T28 and PTU groups is at day 35 pp not different from the continuous hypothyroid animals. It remains to be investigated whether an increase in progenitor Leydig cell proliferation in the first days after discontinuation of the hypothyroid condition occurs. This would be in line with the Sertoli cell data, and explain why transient PTU-induced hypothyroidism leads to a 69% increase in Leydig cell number per testis (Hardy et al., 1993), an expansion in the size of the adult Leydig cell population that is not observed in continuous fetal-postnatal hypothyroidism (Rijntjes et al., 2009). Furthermore, the 4 weeks of reduced plasma TH concentrations have affected the growth and differentiation of the progenitor/immature Leydig cell population in such a way that discontinuation of the condition does not lead to a Leydig cell proliferation profile comparable to that of euthyroid controls; BrdU incorporation remains significantly elevated in the T28 and PTU groups up to 63 days pp, extending the data previously published by Hardy et al. (1996).

The number of studies addressing the consequences of idiopathic hypothyroidism in combination with macroorchidism in prepubertal boys, and its consequences for testicular size and function in adulthood, is extremely limited. (Castro-Magana et al., 1988) reported that these patients had elevated FSH, LH, and prolactin concentrations but normal for age testosterone concentrations. Prepubertal treatment of these patients with TH reduced LH, FSH, and prolactin concentrations within a few months to normal prepubertal levels. Although TH treatment also caused a decrease in testis size, the testis remained significantly larger compared to euthyroid prepubertal children. When these patients reached puberty their testicular function appeared normal, as judged by normal pubertal gonadotropin and testosterone concentrations (Castro-Magana et al., 1988).

Taken together, transient dietary-induced hypothyroidism has various effects on Leydig cell and Sertoli cell development, depending on the time point of discontinuation of the hypothyroid condition. Cessation of the condition up to day 14 pp does not seem to affect the normal development of the Sertoli cell and adult Leydig cell populations as compared to discontinuation of the hypothyroid condition at day 28 pp. We hypothesize that the switch to a euthyroid state leads to enhanced Sertoli cell proliferation, while the formation of the adult-type Leydig cell population is under this condition delayed. This implicates that the increased testis size in adulthood in transiently hypothyroid rats is not caused by the hypothyroid condition *per se*, but originates from augmented Sertoli cell proliferation as a consequence of the rapid normalization of TH concentrations following discontinuation of the diet around day 28 pp.

## Ethics statement

The animal experiments were approved by the Animal Welfare Committee (DEC) of Wageningen University (DEC 2006006, DEC 2008048, DEC 2009059).

## Author contributions

ER participated in designing and performing the experiments, analyzed the data, and took part in writing the manuscript. MLMG, NZ and HJMS participated in performing the experiments. JK facilitated the experiments and critically reviewed the manuscript. KJT was involved in the design of the experiment, helped with the data analysis and writing of the manuscript. All authors read and approved the final manuscript.

## Funding

This project was partially funded by the Deutsche Forschungsgemeinschaft under project RI 2457/1-1 to ER. MLMG was supported by a CAPES/Nuffic 4 scholarship.

### Conflict of interest statement

The authors declare that the research was conducted in the absence of any commercial or financial relationships that could be construed as a potential conflict of interest.

## Supplementary material

Supplementary Figure 1 Immunofluorescent staining for the presence of the androgen receptor (AR) in testis of 28-day-old **(A,B)** and 30-day-old **(C,D)** euthyroid control **(A,C)** and T28 **(B,D)** rats. Formalin fixed, paraffin embedded testis sections were treated as described in the Materials and Methods with minor adaptations. Briefly, following antigen retrieval in sodium citrate buffer (0.1 M, pH 6.0) sections were blocked with 2% normal goat serum (Vector Lab.) and thereafter incubated overnight at 4 C with a primary polyclonal AR antibody (Santa Cruz Biotechn. Inc., Heidelberg, Germany) diluted 1:200 in PBS/BSAc. Next sections were rinsed and incubated for 60 min with a secondary fluorescent goat-anti-rabbit antibody (Alexa Fluor 488, Invitrogen, Eugene, Oregon, USA). Sections were rinsed again, nuclei were stained with Hoechst (Invitrogen) diluted 1:1500 and sections were mounted with Vectashield (Vector Lab.). AR-positive nuclei are stained blue-green, AR-negative nuclei stain blue. AR-positive cells are indicated by arrows (SC, Sertoli cell; LC, Leydig cell; PM, peritubular/myoid cell).

Supplementary Table 1 Plasma thyroid stimulating hormone (TSH; ng/ml) and total thyroxine concentrations (in ng/ml) of euthyroid control, continuously hypothyroid, and transiently (T1, T7, and T14) hypothyroid dams. At the time of dissection TSH and T_4_ concentrations in transiently hypothyroid dams were similar to that of the euthyroid control dams. Values represent means + SEM. ^*^Indicates a significant difference from the euthyroid control dams (*p* < 0.05), *n* = 5–8. ND, non-detectable.

Supplementary Table 2 Body weight, testis weight, plasma thyroid stimulating hormone (TSH), total T_4_, total T_3_, testosterone (Tes.), LH, and FSH concentrations in euthyroid control, continuously hypothyroid, and transiently (T1, T7, and T14) hypothyroid rats from day 12 to 21. Values represent means ± SEM. ^*^Indicates a significant difference from the euthyroid controls (*p* < 0.05), *n* = 5–8. ND, non-detectable.
