# Supplementary figures and images for: Transient Hypothyroidism: Dual Effect on Adult-Type Leydig Cell and Sertoli Cell Development
Source: Front Physiol. 2017 May 23;8:323. doi: 10.3389/fphys.2017.00323 (PMC5441398; doi:10.3389/fphys.2017.00323)

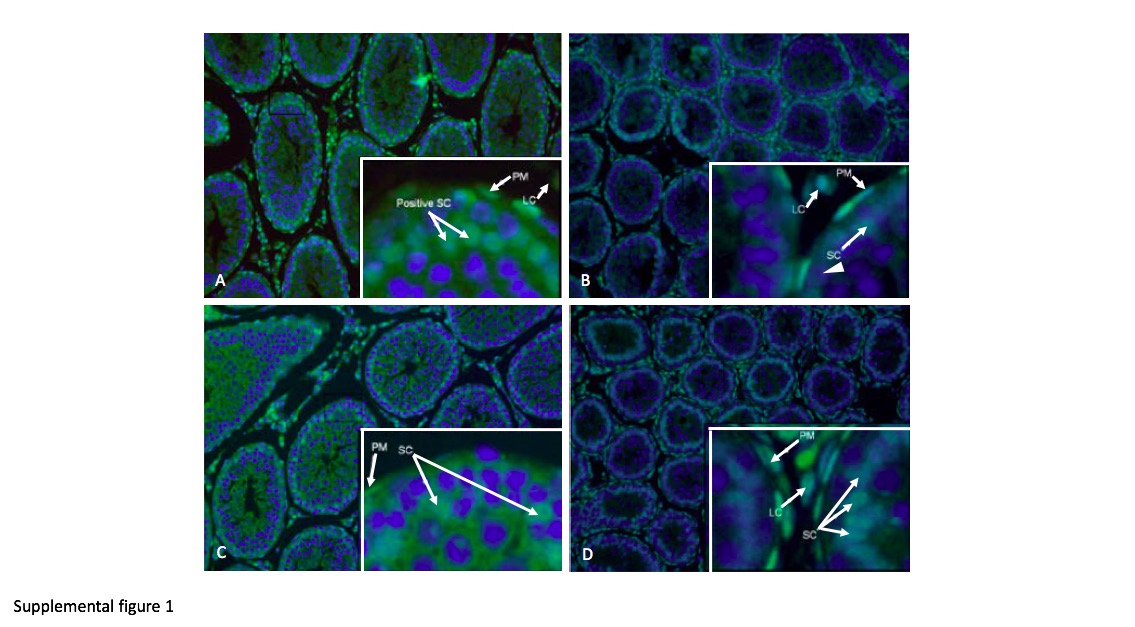

Supplement: Supplementary Figure 1 — Immunofluorescent staining for the presence of the androgen receptor (AR) in testis of 28-day-old (A,B) and 30-day-old (C,D) euthyroid control (A,C) and T28 (B,D) rats. Formalin fixed, paraffin embedded testis sections were treated as described in the Materials and Methods with minor adaptations. Briefly, following antigen retrieval in sodium citrate buffer (0.1 M, pH 6.0) sections were blocked with 2% normal goat serum (Vector Lab.) and thereafter incubated overnight at 4 C with a primary polyclonal AR antibody (Santa Cruz Biotechn. Inc., Heidelberg, Germany) diluted 1:200 in PBS/BSAc. Next sections were rinsed and incubated for 60 min with a secondary fluorescent goat-anti-rabbit antibody (Alexa Fluor 488, Invitrogen, Eugene, Oregon, USA). Sections were rinsed again, nuclei were stained with Hoechst (Invitrogen) diluted 1:1500 and sections were mounted with Vectashield (Vector Lab.). AR-positive nuclei are stained blue-green, AR-negative nuclei stain blue. AR-positive cells are indicated by arrows (SC, Sertoli cell; LC, Leydig cell; PM, peritubular/myoid cell). [file Image1.JPEG]
