# Supplementary material for: Transient Hypothyroidism: Dual Effect on Adult-Type Leydig Cell and Sertoli Cell Development
Source: Front Physiol. 2017 May 23;8:323. doi: 10.3389/fphys.2017.00323 (PMC5441398; doi:10.3389/fphys.2017.00323)
Supplement: Supplementary Table 1 — Plasma thyroid stimulating hormone (TSH; ng/ml) and total thyroxine concentrations (in ng/ml) of euthyroid control, continuously hypothyroid, and transiently (T1, T7, and T14) hypothyroid dams. At the time of dissection TSH and T4 concentrations in transiently hypothyroid dams were similar to that of the euthyroid control dams. Values represent means + SEM. *Indicates a significant difference from the euthyroid control dams (p < 0.05), n = 5–8. ND, non-detectable. [file Table1.DOCX]

Supplemental Table 1. Plasma thyroid stimulating hormone (TSH; ng/ml) and total thyroxine concentrations (in ng/ml) of euthyroid control, continuously hypothyroid and transiently (T1, T7 and T14) hypothyroid dams. At the time of dissection TSH and T_4_ concentrations in transiently hypothyroid dams were similar to that of the euthyroid control dams. Values represent means + SEM. * indicates a significant difference from the euthyroid control dams (p<0.05), n=5-8. ND: non-detectable.

| Dams | Control | Hypothyroid | T1 | T7 | T14 |
| --- | --- | --- | --- | --- | --- |
| TSH (ng/ml)  -conception  -parturition  -weaning | 0.65 ± 0.05  0.76 ± 0.09  0.87 ± 0.09 | 10.21* ± 0.38  28.10* ± 2.85  21.42* ± 1.24 | 0.45 ± 0.11 | 0.56 ± 0.07 | 0.44 ± 0.08 |
| T_4_ (ng/ml)  -conception  -parturition  -weaning | 34 ± 3  24 ± 1  31 ± 3 | 5* ± 1  ND  ND | 24 ± 3 | 25 ± 3 | 28 ± 3 |
