# Supplementary material for: Transient Hypothyroidism: Dual Effect on Adult-Type Leydig Cell and Sertoli Cell Development
Source: Front Physiol. 2017 May 23;8:323. doi: 10.3389/fphys.2017.00323 (PMC5441398; doi:10.3389/fphys.2017.00323)
Supplement: Supplementary Table 2 — Body weight, testis weight, plasma thyroid stimulating hormone (TSH), total T4, total T3, testosterone (Tes.), LH, and FSH concentrations in euthyroid control, continuously hypothyroid, and transiently (T1, T7, and T14) hypothyroid rats from day 12 to 21. Values represent means ± SEM. *Indicates a significant difference from the euthyroid controls (p < 0.05), n = 5–8. ND, non-detectable. [file Table2.DOCX]

Supplemental Table 2. Body weight, testis weight, plasma thyroid stimulating hormone (TSH), total T_4_, total T_3_, testosterone (Tes.), LH and FSH concentrations in euthyroid control, continuously hypothyroid and transiently (T1, T7 and T14) hypothyroid rats from day 12 to 21. Values represent means ± SEM. * indicates a significant difference from the euthyroid controls (p<0.05), n=5-8. ND: non-detectable.

| Offspring | Control | Hypothyroid | T1 | T7 | T14 |
| --- | --- | --- | --- | --- | --- |
| Day 12  BW (g)  TW (mg)  TSH (ng/ml)  T_4_ (ng/ml)  T_3_ (ng/ml) | 22.5 ± 2.20  24 ± 3  0.21 ± 0.06  24 ± 8  1.6 ± 0.20 | 16.6 ± 2.40  21 ± 6  4.13* ± 1.94  1* ± 1  1.7 ± 0.0 | 22.2 ± 0.50  20 ± 0  0.48 ± 0.10  16 ± 1  1.7 ± 0.10 |  |  |
| Day 16  BW (g)  TW (mg)  TSH (ng/ml)  T_4_ (ng/ml)  T_3_ (ng/ml)  Tes. (ng/ml)  LH (ng/ml)  FSH (ng/ml) | 29.6 ± 1.80  40 ± 3  0.26 ± 0.04  40 ± 3  2.1 ± 0.10  0.99 ± 0.07  0.35 ± 0.07  6 ± 1 | 23.0* ± 1.50  31 ± 1  11.94* ± 0.88  ND  1.8 ± 0.10  0.64 ± 0.08  0.37 ± 0.25  4 ± 1 | 29.4 ± 1.20  37 ± 3  0.29 ± 0.06  39 ± 7  1.9 ± 0.20  0.84 ± 0.22  0.35 ± 0.14  5 ± 1 | 27.2 ± 1.70  35 ± 3  0.45 ± 0.15  29 ± 5  1.9 ± 0.30  0.67 ± 0.19  0.15 ± 0.02  5 ± 1 |  |
| Day 21  BW (g)  TW (mg)  TSH (ng/ml)  T_4_ (ng/ml)  T_3_ (ng/ml)  Tes. (ng/ml)  LH (ng/ml)  FSH (ng/ml) | 41.7 ± 1.80  80 ± 6  0.39 ± 0.21  34 ± 3  2.1 ± 0.10  0.64 ± 0.10  1.83 ± 0.36  10 ± 1 | 30.5* ± 1.80  51* ± 4  11.60* ± 2.70  5* ± 5  1.7 ± 0.20  1.35* ± 0.21  0.46* ± 0.07  4* ± 0 | 44.0 ± 4.10  82 ± 11  0.55 ± 0.09  29 ± 4  2.0 ± 0.0  0.89 ± 0.14  1.27 ± 0.29  9 ± 1 | 43.4 ± 4.00  81 ± 8  0.30 ± 0.05  27 ± 3  1.7 ± 0.20  0.79 ± 0.19  1.06 ± 0.42  7 ± 1 | 36.9 ± 1.60  70 ± 6  0.71 ± 0.15  27 ± 3  1.7 ± 0.10  0.90 ± 0.11  0.72 ± 0.14  7 ± 1 |
